# Mutations in the proximal binding site and F-loop of AdeJ confer resistance to efflux pump inhibitors

**DOI:** 10.1128/aac.00090-25

**Published:** 2025-07-08

**Authors:** Aysegul Saral Sariyer, Inga V. Leus, Rushikesh Tambat, Mithila Farjana, Marcela Olvera, Shalini J. Rukmani, Emrah Sariyer, Jeremy C. Smith, Jerry M. Parks, John K. Walker, Helen I. Zgurskaya

**Affiliations:** 1 Department of Chemistry and Biochemistry, University of Oklahoma 522128 , Norman, Oklahoma, USA; 2 Department of Nutrition and Dietetics, Faculty of Health Sciences, Artvin Coruh University 187431, Artvin, Turkey; 3 Biosciences Division, Oak Ridge National Laboratory 6146, Oak Ridge, Tennessee, USA; 4 Vocational School of Health Services, Medical Laboratory Techniques, Artvin Coruh University 187431, Artvin, Turkey; 5 Department of Pharmacology and Physiology, School of Medicine, Saint Louis University 12274, St. Louis, Missouri, USA; 6 Department of Chemistry, Saint Louis University 219030, St. Louis, Missouri, USA; Shionogi Inc., Florham Park, New Jersey, USA

**Keywords:** *Acinetobacter*, antibiotic resistance, multidrug efflux, efflux pump inhibitors

## Abstract

Multidrug efflux is one of the major mechanisms of antibiotic resistance in gram-negative bacteria. Inhibitors of efflux pumps potentiate the activities of antibiotics, and their discovery could lead to new therapeutic options. The AdeIJK pump in *Acinetobacter baumannii* is a promising target for efflux pump inhibitors (EPIs) due to its high clinical importance and conservation, and several classes of EPIs targeting this and other *A. baumannii* efflux transporters have been recently reported. However, the mechanisms of action of these EPIs and their resistance liability remain underexplored. Here, we analyzed the impact of site-specific substitutions in the substrate/EPI translocation path of the inner membrane transporter AdeJ on efflux of substrate antibiotics and fluorescent probes and activities of substituted 4,6-diaminoquinoline EPIs. We found that substitutions in amino acid residues located in the entrance cleft (R701) and the flexible loop (E675) of AdeJ lead to resistance specifically against biphenyl-substituted EPIs, whereas the substitution of F178 in the distal binding pocket increased AdeJ sensitivity to certain naphthyl- and biphenyl-substituted EPIs. No major differences in docking scores and poses of substrates and EPIs were observed between the wild type and corresponding AdeJ variants for any of the mutations considered. This study concludes that substrates and EPIs bound along the translocation path of AdeJ participate in its conformational transitions and can either increase or decrease the rate of transport and therefore the efficiency of EPIs.

## INTRODUCTION

*Acinetobacter baumannii* is an opportunistic, gram-negative bacterium that can cause severe infections in humans that are challenging to cure. The prevalence of *A. baumannii* infections has been rising annually, and it is widely recognized as a leading source of nosocomial infections. *A. baumannii* is currently considered a health priority for additional research and development by the World Health Organization and the Centers for Disease Control and Prevention (1, 2).

*A. baumannii* and other gram-negative bacteria use a variety of special defense mechanisms to resist antibiotics. Among these, the overexpression of efflux pumps, the reduction in outer membrane (OM) permeability, the expression of antibiotic-modifying enzymes, and target mutations are the prevalent resistance mechanisms (3). Indeed, in *A. baumannii,* overexpression of efflux pumps belonging to the resistance nodulation division (RND) superfamily is the main mechanism of clinically relevant multidrug resistance (MDR) (4). Six RND efflux pumps have been characterized to varying degrees in *Acinetobacter* spp., and the role of these pumps in *A. baumannii* MDR is being investigated (5). AdeIJK, a constitutively expressed multidrug efflux pump, is the only RND pump that has been discovered in all *A. baumannii* strains to date, suggesting that the bacterial life cycle depends on its physiological function (5, 6).

AdeIJK plays a major role in the intrinsic antibiotic resistance observed in *A. baumannii*. This pump has been found to effectively efflux out a plethora of antibiotics belonging to different chemical classes such as tetracyclines, phenicols, macrolides, triclosan, fluoroquinolones, trimethoprim, and beta-lactams (5, 7, 8). Recent studies have shown that deletion of AdeIJK leads not only to hypersusceptibility to antibiotics (9) but also to significant changes in gene expression (10), confirming that the pump is tightly integrated into *A. baumannii* physiology.

In AdeIJK, the inner membrane-based RND transporter, AdeJ associates with the outer membrane channel, AdeK of the outer membrane factor family, as well as the periplasmic adaptor protein, AdeI of the membrane fusion protein family (11). The tripartite nature of AdeIJK and other RND-type pumps provides machinery enabling them to translocate substrates across the entire cell envelope while hindering the drugs from reentering cells through the low-permeability outer membrane (11, 12). For the AdeJ transporter to bind and expel substrates, each of its protomers must cycle through a minimum of three conformational states, referred to as access, binding, and extrusion (13–16). The distal binding pocket (DBP) and the proximal binding pocket (PBP) are two unique binding pockets to which substrates and inhibitors bind. A short loop (F-loop) sits in between the pockets and divides them and is thought to be important during the access to binding conformational state change (17, 18). Substrates attach to the distal pocket after accessing the proximal binding site (18). Structures of AdeJ in ligand-bound states are available and show that the drugs eravacycline (ERV) and fluorocycline TP-6076 engage in hydrophobic interactions with V139, F178, G179, F277, A326, Y327, F611, V613, F616, and F629 in the DBP of AdeJ (14).

The development of efflux pump inhibitors (EPIs) offers a promising approach to resensitize bacteria to antibiotics, potentially enhancing the longevity and effectiveness of existing antibiotic treatments (19–21). The best-characterized EPIs targeting RND pumps, such as the pyridopyrimidine-class inhibitors of MexAB-OprM in *Pseudomonas aeruginosa* and the pyranopyridine-based inhibitors of AcrAB-TolC in *Escherichia coli*, were found to bind to the DBP, specifically to the patch of aromatic residues called the hydrophobic trap. These EPIs share binding residues with known substrates of these efflux pumps and are thought to inhibit efflux by slowing down the conformational cycling of the RND transporters (22–24). More recently, computational and experimental analyses of MexAB-OprM inhibitors suggested that interactions of substrates and EPIs with the target MexB differ at the interface between the PBP and DBP and that the two types of ligands therefore engage different residues for efficient efflux and its inhibition, respectively (25). Molecular dynamics simulation studies of AcrAB-TolC and MexAB-OprM and their inhibitors have found that in these systems, the distal sites bind substrates more tightly than the proximal and entrance sites (25, 26). Furthermore, it was found for AcrAB-TolC that antibiotic resistance does not always correlate with higher binding affinities for compounds but is largely dependent on conformational changes, especially those associated with the flexibility of the switch loops between the DBP and PBP (23, 27, 28).

The AdeIJK pump in *A. baumannii* is a promising target for EPIs due to its high clinical importance and conservation, and a few classes of EPIs targeting *A. baumannii* RND transporters have been reported recently (29, 30). We previously found that compounds from the 4,6-diaminoquinoline and 4-dihydroimidazoylaniline families of EPIs specifically inhibit the AdeIJK pump and potentiate the activities of antibiotics, including erythromycin (ERY), novobiocin (NOV), and tetracycline (TET), in antibiotic-susceptible *A. baumannii* American Type Culture Collection (ATCC) 17978 and the clinical multidrug-resistant isolates AB5075 and AYE, which overproduce both AdeIJK and AdeABC pumps (10, 30). These inhibitors potentiate the activities of the antibiotics in the wild type (WT) and AdeIJK-overproducing cells and their derivatives with hyperporinated OM but not in the AdeJ-deficient backgrounds, suggesting a specific anti-AdeIJK activity independent of the permeability across the OM. They also do not act as membrane uncouplers. In this study, we introduced mutations into the putative substrate/inhibitor translocation route of AdeJ and analyzed the effect of these mutations on efflux activity and the potency of the inhibitors. We unexpectedly found that mutations in the access binding site led to resistance to EPIs. The resistance is associated with the chemical structure of the inhibitor, suggesting a direct effect on inhibitor binding. Our results suggest that AdeJ-specific inhibitors could act on the pump by a new mechanism that involves binding to the access pocket and slowing conformational transitions of AdeJ.

## RESULTS

### Substitutions in the substrate translocation path of AdeJ have an antibiotic-specific effect on *A. baumannii* susceptibility

**Fig 1:**
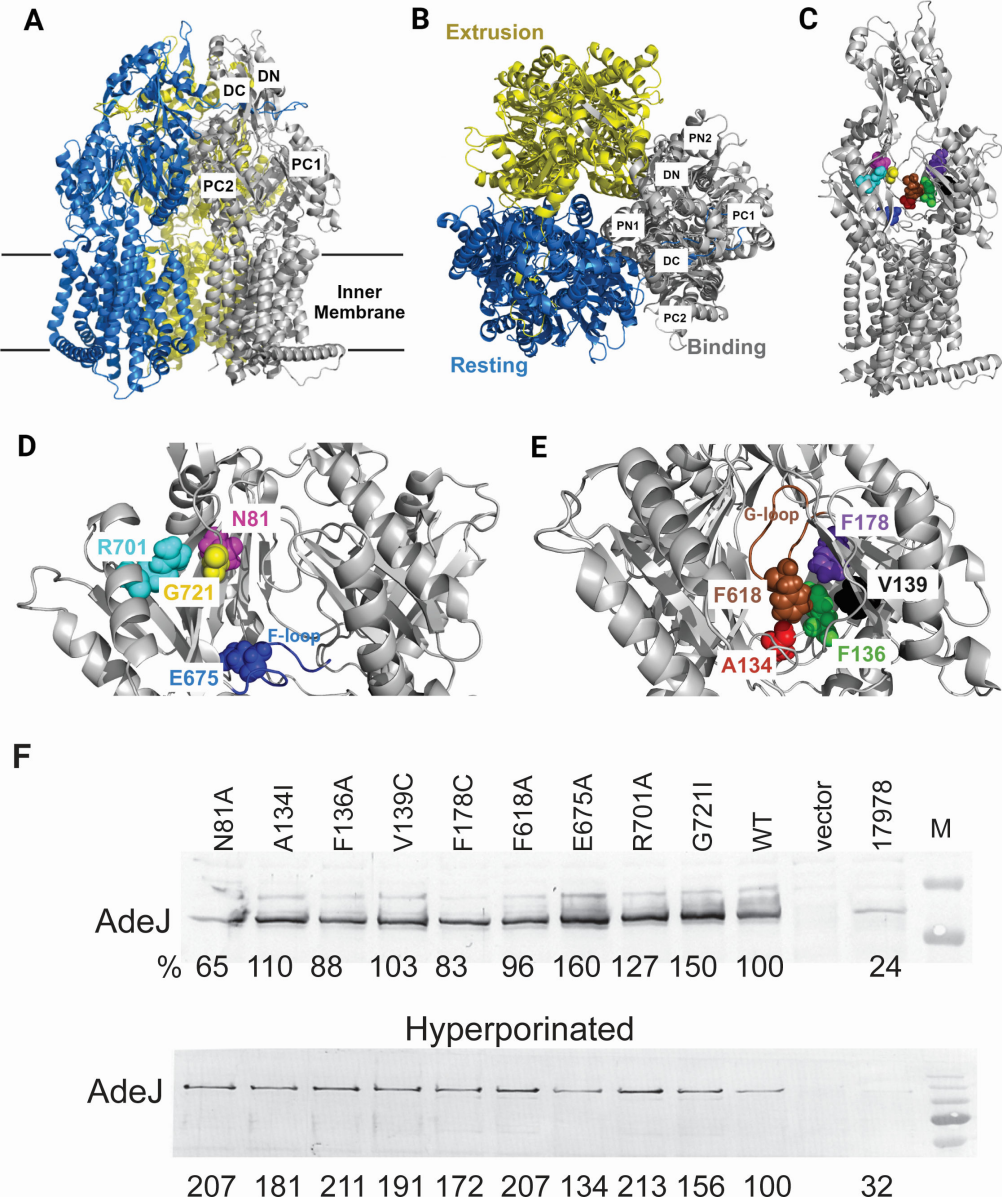
Structure of AdeJ, amino acid residues mutated in this study, and the expression of the constructed AdeJ variants. (**A**) Side and (**B**) top views of the cryo-electron microscopy structure of trimeric AdeJ in the apo state (PDB ID: 7M4Q [31]). (**C**) Binding monomer of AdeJ with the substituted amino acid residues shown as spheres. (**D**) Close view of residues in the entrance and proximal binding pocket and the F-loop. (**E**) Close view of residues in the distal binding pocket and the G-loop. Created in BioRender. (**F**) Immunoblotting analysis of cell lysates isolated from the wild type (17978) and efflux-deficient AbΔ3 cells carrying an empty vector and the indicated plasmid-borne AdeJ variants (top) and corresponding hyperporinated derivatives (bottom). AdeJ variants were visualized with an anti-AdeJ polyclonal antibody.

Structural and computational studies have suggested that substrates entering the drug-binding site of AdeJ are guided by the F-loop to travel to the PBP and then pass through the gate loop (G-loop) and be delivered to the DBP for extrusion (31). We constructed nine AdeJ mutants containing amino acid substitutions in the PBP of AdeJ (G721I, R701A, and N81A), F-loop (E675A), G-loop (F618A), and the DBP (F136A, F178C, A134I, and V139C) (Fig. 1). All constructed mutants were expressed at similar within twofold levels and well above the native AdeJ levels in ATCC 17978 (Fig. 1F; Table S1).

To analyze the functionality of the constructed AdeJ variants, we first measured minimal inhibitory concentrations (MICs) of antibiotics that are known substrates of AdeJ. The MICs were measured in the efflux-deficient *A. baumannii* strain AbΔ3 (Δ*adeIJK ΔadeAB* Δade*FGH*), which lacks the three most common RND efflux pumps (Table 1), and its hyperporinated variant AbΔ3-pore, which produces a large non-specific pore in the outer membrane (Table S2) carrying the constructed plasmid-borne AdeIJK variants. We found that the AdeJ F178C variant with a substitution in the DBP was notably less functional than other constructs, as seen from the reduced MICs of multiple substrates such as sodium dodecyl sulfate (SDS), ethidium bromide (EtBr), and NOV. The analogous substitution in homologous transporters resulted either in reduced or enhanced transport activity, depending on the transporter and substrate (25, 32). In addition, cells producing AdeJ V139C, E675A, and G721I variants were more susceptible than WT only to SDS.

**TABLE 1:** Minimal inhibitory concentrations (MICs) of antibiotics in the wild type, efflux-deficient *A. baumannii* strains, and the efflux-deficient strain producing the indicated AdeJ variants^*a*^

Strains	Location	MICs (µg/mL)									
		ERY	NOV	ZEO	CIP	NOR	ERV	TET	CHL	SDS	EtBr
Vector	NA	2.5	0.04	2.0	0.016	0.125	0.016	0.016	16.0	32	<0.5
WNAT	NA	10	4.0	16.0	0.125	4.0	0.125	0.25	64.0	>1,024	128.0
E675A	F-loop	20	8.0	32.0	0.25	8.0	0.25	0.25	64.0	512	64.0
N81A	PBP	20	4.0	16.0	0.25	8.0	0.5	0.5	128.0	>1,024	256.0
R701A		20	8.0	16.0	0.5	8.0	0.25	0.5	128.0	>1,024	256.0
G721I		20	8.0	8.0	0.25	8.0	0.25	0.25	128.0	>1,024	128.0
F618A	G-loop	20	4.0	8.0	0.5	8.0	0.25	0.5	256.0	>1,024	128.0
A134I	DBP	10	4.0	8.0	0.25	8.0	0.25	1.0	128.0	>1,024	256.0
F136A		20	4.0	8.0	0.5	16.0	0.5	1.0	256.0	>1,024	256.0
V139C		10	4.0	16.0	0.25	4.0	0.25	0.25	128.0	512	64.0
F178C		10	1.0	32.0	0.25	4.0	0.125	0.25	32.0	256	16.0

^*a*^ :CHL, chloramphenicol; CIP, ciprofloxacin; DBP, distal binding pocket; ERV, eravacycline; ERY, erythromycin; EtBr, ethidium bromide; NOR, norfloxacin; NOV, novobiocin; PBP, proximal binding pocket; SDS, sodium dodecyl sulfate; TET, tetracycline; ZEO, zeocin; NA, not applicable.

Four AdeJ variants (N81A, A134I, F136A, and R701A) enabled a modest increase of two to four in MICs of ERY and EtBr (Table 1). In addition, F136A also positively affected the efflux of TET, ERV, and chloramphenicol (CHL). Thus, mutations modify the activities of only some substrates and in a site-specific manner.

### AdeJ mutant variants vary in bacterial growth-independent efflux of fluorescent substrates

**Fig 2:**
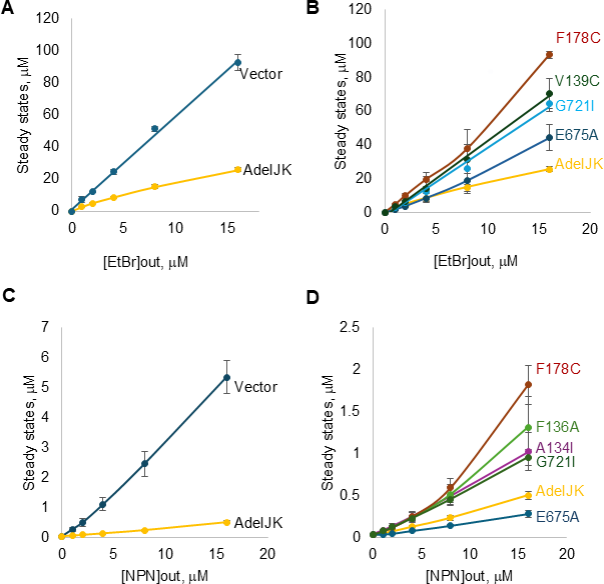
Steady-state accumulation levels of the fluorescent substrates EtBr and NPN in cells producing AdeIJK and its variants. (**A and B**) Concentration-dependent accumulation levels of Et in *A. baumannii* cells carrying an empty vector, WT AdeIJK, and its mutated variants. (**C and D**) The same as panels A and B, but NPN was used as a substrate. Cells were incubated with indicated increasing concentrations of substrates (out), and real-time fluorescence was monitored. Kinetic curves (Fig. S1 and S2) were fitted to a two-exponential model, and the calculated steady-state accumulation levels were plotted as a function of the external concentration (out) of each substrate.

We next analyzed efflux activities of the constructed AdeJ mutants in bacterial growth-independent assays. For this purpose, we used two fluorescent probes, EtBr and *N*-phenyl-naphthylamine (NPN), which were previously identified as substrates of the AdeIJK pump. We analyzed the kinetics of the intracellular accumulation of EtBr and NPN in AbΔ3 and AbΔ3-pore cells, respectively, producing the constructed AdeIJK variants (Fig. 2; Fig. S1 and S2). These two probes have low fluorescence in water, which is notably enhanced when EtBr is bound to supercoiled DNA or when NPN is intercalated into membranes. Hence, they report on the concentrations of the respective probe in the *A. baumannii* cytoplasm and the cellular membranes, respectively. The collected time-dependent changes in fluorescence of probes were fitted using a model to extract their steady-state intracellular concentrations (33).

According to MICs, only cells producing F178C were more susceptible to EtBr, whereas several mutants were somewhat more efficient in efflux of EtBr than the WT (Table S2). In agreement, the F178C variant failed to prevent the accumulation of EtBr in cells, and the steady-state levels of EtBr in these cells were like those in the cells carrying an empty vector (Fig. 2A and B). In addition, V139C, G721I, and less so E675A were partially defective in the efflux of EtBr. Activities of all the other mutants matched that of the WT pump.

All AdeJ mutants were able to expel NPN (Fig. 2C and D). However, F178C again was the most defective, followed by F136A, A134I, and G721I, with partial loss of efflux. In contrast, the E675A mutant was more efficient than WT in efflux of NPN. Thus, in agreement with MICs, F178C led to a non-specific loss of activity, whereas the effect of other substitutions is specific to substrates.

### Substitutions in the F-loop and proximal binding pocket lead to resistance against EPIs

**Fig 3:**
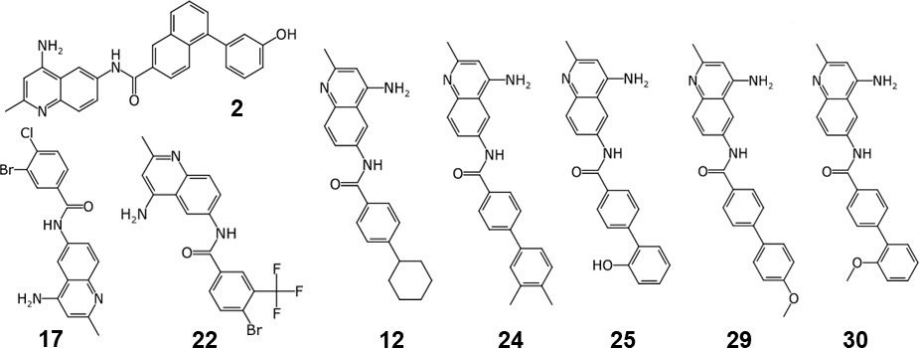
Chemical structures of EPIs used in this study.

We previously identified AdeIJK-specific EPIs, and based on their properties, we proposed that they are likely to act by binding to the hydrophobic trap in the DBP of AdeJ (30). Here we analyzed how activities of the EPIs are affected by site-specific mutations in AdeJ. We focused on eight of our most active compounds from across three differently substituted 4,6-diaminoquinolines (DAQ). We used a representative naphthyl-substituted DAQ analog, **2,** benzoyl-substituted DAQs (**12**, **17**, and **22**), and biphenyl-substituted DAQs (**24**, **25**, **29**, and **30**) (Fig. 3). In agreement with previous studies which showed that these EPIs are substrates of AdeIJK (30), all compounds, except **29**, inhibited growth of the efflux-deficient AbΔ3-pore with MICs ranging between 12.5 and 200.0 µM (Table 2). However, only compounds **12** and **22** had notable antibacterial activities against AbΔ3(AdeIJK)-pore, both with MICs = 50 µM, which are still fourfold higher than MICs of these compounds in AbΔ3-pore. Substitutions in AdeJ did not change the MICs of compounds by more than twofold (Table 2).

**TABLE 2:** MICs of efflux pump inhibitors in *A. baumannii* Δ3-pore cells carrying plasmids producing AdeIJK with indicated AdeJ variants

Strains	MICs (µM)							
	2	12	17	22	24	25	29	30
Vector	25	12.5	50	12.5	50	100	>200	12.5
WT	>200	50	200	50	>200	200	>200	>200
E675A	>200	25	>200	100	>200	100	>200	>200
N81A	>200	25	100	50	200	100	>200	>200
R701A	>200	100	200	50	>200	100	>200	>200
G721I	>200	25	200	50	>200	100	>200	>200
F618A	>200	50	200	100	>200	100	>200	>200
A134I	>200	100	>200	100	>200	100	>200	>200
F136A	>200	25	200	50	200	100	>200	>200
V139C	>200	50	200	100	>200	100	>200	>200
F178C	>200	25	100	50	50	100	>200	100

To analyze the effect of mutations in AdeJ, we first measured minimal potentiating concentrations of inhibitors that reduce the MICs of the antibiotics NOV, ERY, and TET, the AdeJ substrates, by at least fourfold (MPC_4_). In agreement with previous findings, WT AdeIJK was inhibited by all eight compounds, with MPC_4_ values ranging from 3.125 to 12.5 μM in combination with NOV and from 6.25 to 25.0 μM in combination with ERY (Table 3). The potentiation of antibacterial activity of EtBr and TET was weaker, with MPC_4_ values ranging from 12.5 to 50.0 μM, depending on the compound. None of the substitutions in AdeIJK affected MPC_4_ values of the benzoyl-substituted DAQs, **12**, **17**, and **22** (Table S3). However, F178C, E675A, and R701A substitutions in AdeJ notably changed the sensitivity to other series of EPIs. In combination with tested antibiotics (Table 3), the F178C variant was 8- to 16-fold more susceptible to naphthyl-substituted **2** and biphenyl-substituted DAQs **29** and **30** containing a 2- and 4-substituted methoxy group in the second phenyl ring, respectively, but not to compound **25** in which the 2-methoxy is changed to a 2-hydroxy substituent, or compound **24**, which contains a 3,4-dimethyl-substituted biphenyl DAQ (Table 3). Thus, not only the size of compounds but also the specific positions of substituents are important for inhibition, and subtle steric issues might be contributing to differences in the activities of these EPIs.

**TABLE 3:** Minimal potentiating concentrations (MPC_4_) of EPIs in combination with indicated antibiotics in Ab∆3-pore strains producing the indicated AdeJ variants^*a*^

Strains	NOV MPC_4_ (µM)				
	Compound 2	Compound 24	Compound 25	Compound 29	Compound 30
WT	12.5	3.125	12.5	6.25	3.125
E675A	12.5	**25.0**	**50.0**	**50.0**	**25.0**
N81A	25	25.0	25.0	12.5	6.25
R701A	12.5	12.5	25.0	6.25	6.25
G721I	12.5	12.5	25.0	6.25	12.5
F618A	12.5	12.5	25.0	6.25	12.5
A134I	12.5	6.25	12.5	6.25	6.25
F136A	25.0	12.5	25.0	25	12.5
V139C	12.5	12.5	12.5	3.125	6.25
F178C	**1.56**	3.125	12.5	**0.78**	**0.78**
	**ERY MPC_4_ (µM**)				
WT	12.5	12.5	12.5	6.25	25.0
E675A	12.5	**50.0**	**50.0**	**50.0**	**100.0**
N81A	12.5	6.25	12.5	6.25	12.5
R701A	12.5	12.5	12.5	**50.0**	**100.0**
G721I	12.5	12.5	12.5	6.25	25.0
F618A	12.5	12.5	12.5	6.25	25.0
A134I	25.0	25.0	25.0	12.5	50.0
F136A	12.5	12.5	6.25	6.25	12.5
V139C	12.5	12.5	12.5	6.25	25.0
F178C	**3.125**	12.5	12.5	**1.56**	**3.125**
	**TET MPC_4_ (µM**)				
WT	50.0	50	25.0	25.0	50.0
E675A	50.0	**100**	**100.0**	**100.0**	**>200.0**
N81A	50.0	50	25.0	25.0	50.0
R701A	50.0	50	25.0	**100.0**	**100.0**
G721I	50.0	50	25.0	25.0	50.0
F618A	50.0	50	25.0	25.0	50.0
A134I	50.0	50	25.0	25.0	50.0
F136A	25.0	25	12.5	12.5	25.0
V139C	50.0	50	25.0	25.0	50.0
F178C	**12.5**	50	25.0	**6.25**	**12.5**

^*a*^ Values in bold are significantly different from the WT.

Zeocin is a large antibiotic which does not readily penetrate the OM of *A. baumannii* and for which the negative effect of F178C substitution is seen in the hyperporinated (Table S2) but not in the cells with native OM (Table 1). Therefore, we also tested whether F178C substitutions lead to increased sensitivity to **2**, **29**, and **30** in the combination with zeocin. Consistent with our previous findings that these EPIs readily penetrate the OM of *A. baumannii* (30)*,* we found that the increased sensitivity of the AdeJ F178C variant to EPIs is seen in both AbΔ3(AdeIJK) with native OM and its hyperporinated derivative (Table S4). This result suggests that the F178C mutation makes AdeJ more sensitive to the EPIs in a substrate-independent manner.

In contrast, E675A became resistant to all biphenyls but not to naphthyl- or phenyl-substituted analogs (Table 3; Table S4), suggesting that interactions with E675 are different from interactions with F178. The resistance of AdeJ E675A was observed with all tested substrates, suggesting that interactions between the EPIs and AdeJ and their corresponding inhibitory activities are independent of the potentiated substrate. However, the properties of the AdeJ R701A variant were different. This variant became resistant only against the activities of compounds **29** and **30** and only in combination with ERY, TET, and zeocin, but not NOV. Thus, unlike F178C and E675A, the effect of the R701A substitution is substrate dependent.

**Fig 4:**
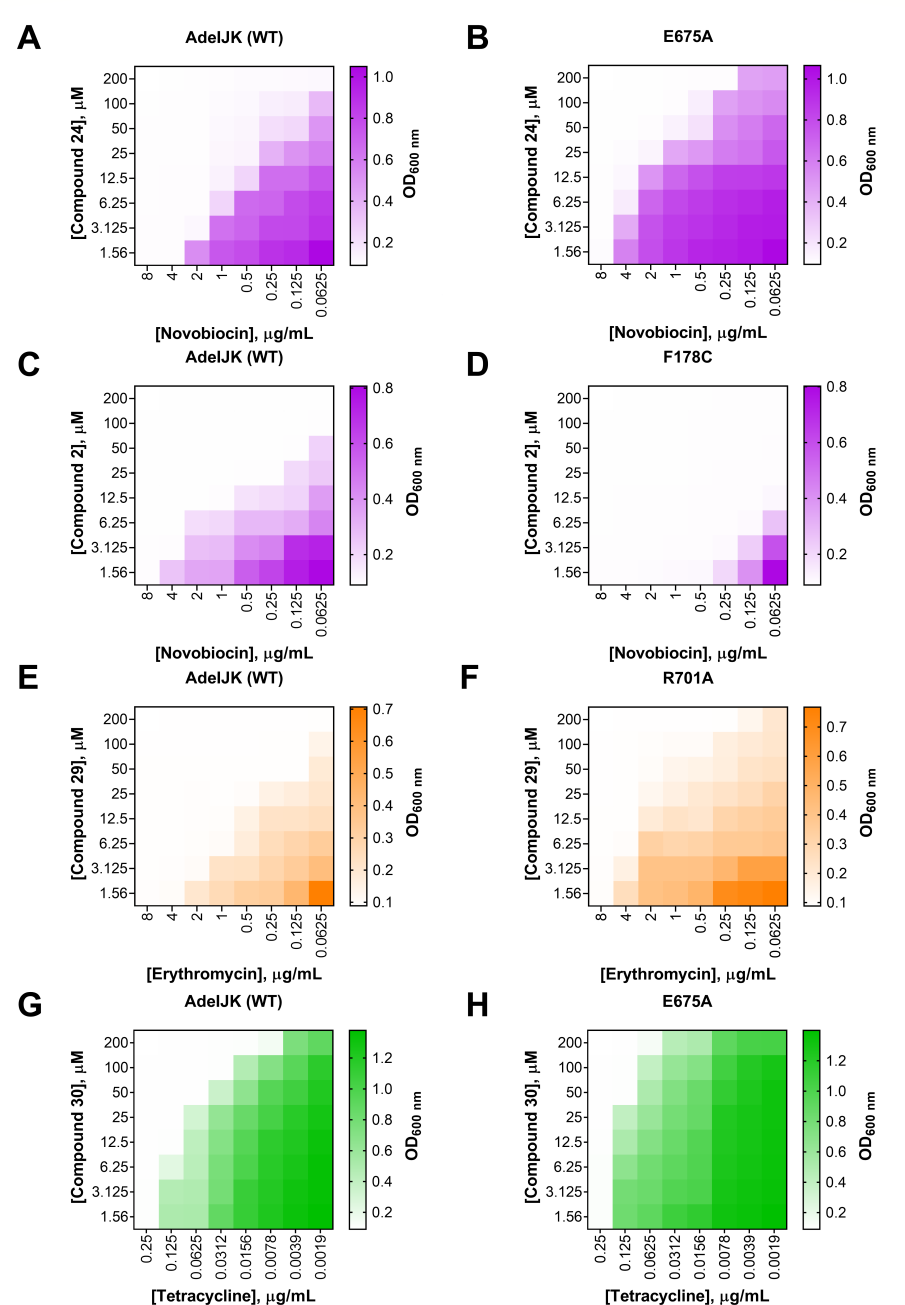
Checkerboard assays with *A. baumannii* AbΔ3-pore cells producing the AdeJ WT, E675A, F178C, and R701A variants for the following combinations: (**A and B**) NOV and the EPI 25, (**C and D**) NOV and the EPI 2, (**E and F**) ERY and the EPI 29, and (**G and H**) TET and the EPI 30.

To determine whether the effects of the mutations in AdeJ were additive or multiplicative, we analyzed the potentiating activities of representative EPIs and antibiotics in a checkerboard assay (Fig. 4; Table S5). We found that when used against the AdeJ E675A and R701A variants, the combinations of NOV with compound **24**, ERY with compound **29**, and TET with compound **30**, respectively, became antagonistic. In contrast, the combination of NOV with compound **2** was highly synergistic against the AdeJ F178C variant.

**Fig 5:**
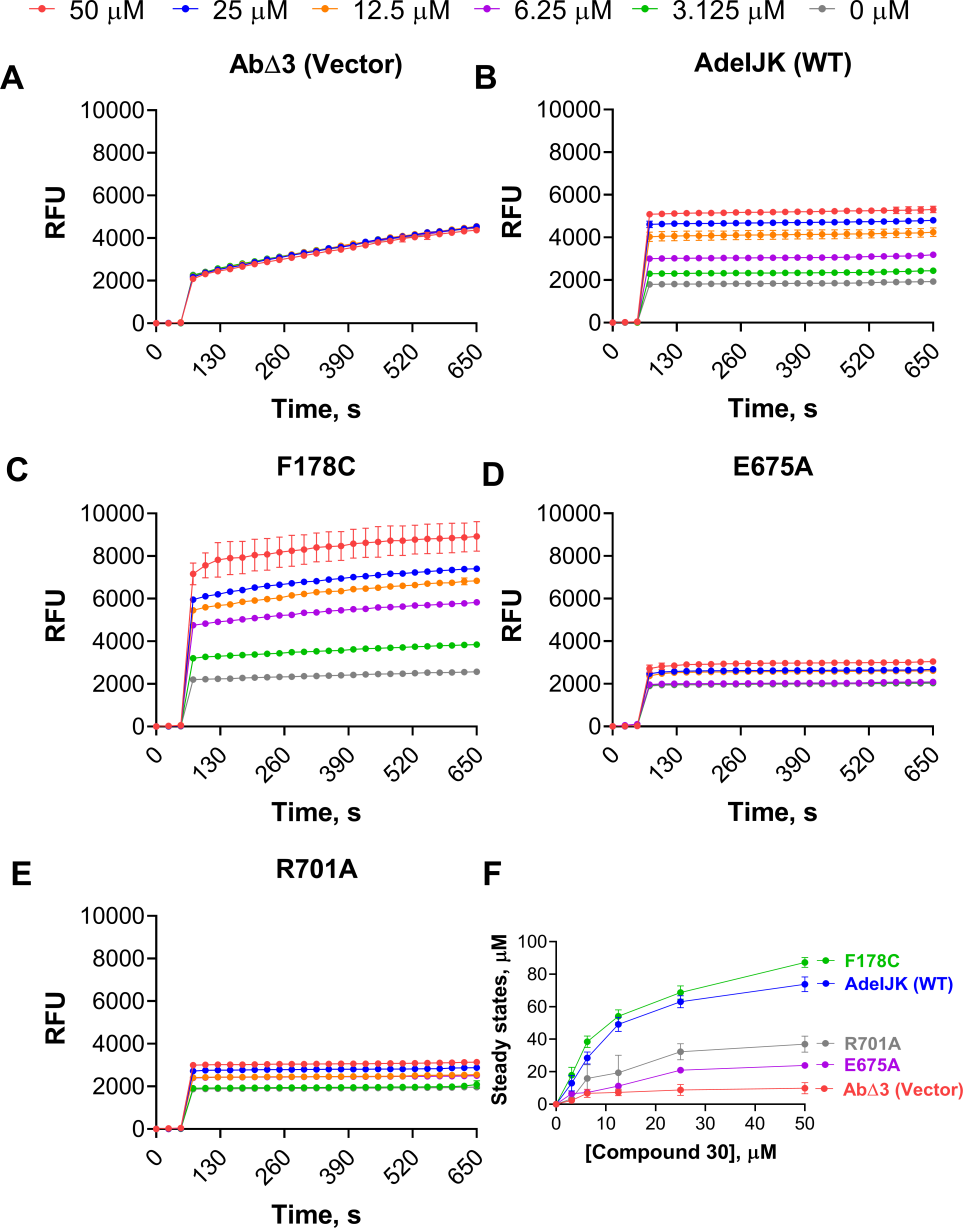
Kinetic curves and steady-state accumulation levels of EtBr in AbΔ3-pore cells carrying empty vector, WT, F178C, E675A, and R701A AdeJ variants. (**A–E**) Kinetic curves of EtBr accumulation in the indicated cells pre-treated with increasing concentrations of compound 30 (shown at the top) and at the constant 5 µM final concentration of EtBr. Relative fluorescence units (RFUs) were calculated by normalizing fluorescence intensity after subtracting background fluorescence without cells and plotted against time. (**F**) Steady-state accumulation levels calculated from the kinetic curves are shown in **A–E**.

The potentiating activity of EPIs in combination with the fluorescent substrate EtBr is also affected by F178C, E675A, and R701A substitutions in AdeJ (Table S4). We next analyzed how these mutations affected the activity of EPIs in the bacterial growth-independent efflux of EtBr (30). For this purpose, cells producing AdeJ WT, F178C, E675A, or R701A variants were incubated with 5 µM EtBr and increasing concentrations of the analogs **2**, **24**, **29**, and **30**. If an EPI inhibits specifically the activity of the pump, we expect to see increasing accumulation of EtBr in cells treated with the EPI in efflux-proficient cells but not in cells lacking AdeJ. As expected, in AbΔ3(AdeIJK)-pore cells treated with the EPIs, EtBr accumulated at higher intracellular steady-state levels and in a concentration-dependent manner (Fig. 5B; Fig. S3). In contrast, no changes in the EtBr accumulation could be seen in cells AbΔ3-pore cells carrying an empty vector, suggesting that the changes in accumulation are due to the inhibition of EtBr efflux by AdeIJK (Fig. 5A; Fig. S3).

In agreement with the substrate potentiation assays, cells producing E675A and R701A variants that are resistant to potentiating activity of EPIs showed very minor changes in EtBr levels even at the highest concentrations of compound **30** (Fig. 5D and E). Likewise, the analogs **24** and **29** had only a weak effect on the intracellular accumulation of EtBr in cells producing E675A and R701A variants, respectively (Fig. S3). In contrast, the F178C variant was effectively inhibited by compound **30**, as seen from the increasing EtBr steady-state levels (Fig. 5C). Similar results were observed with analog **2**, which is highly effective against the F178C variant in the potentiation of antibiotic activities (Fig. S3). We conclude that changes in the potentiating activities of these EPIs are due to the changes in the properties of the AdeJ transporter that are caused by specific amino acid substitutions.

### Substitutions in AdeJ could affect transporter dynamics

**Fig 6:**
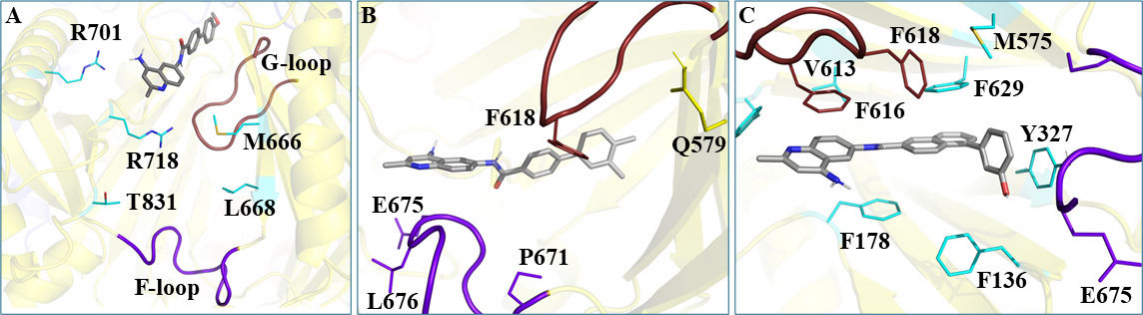
Representative poses of three selected EPIs docked to the binding protomer of AdeJ [PDB ID 7RY3(19), chain C] at three sites. (**A**) Compound 29, bound at the entrance site of AdeJ (score: −8.4 kcal/mol); (**B**) compound 24, bound at the proximal site of the binding protomer (−10.4 kcal/mol); and (**C**) compound 2, bound at the distal site of the binding protomer (−13.2 kcal/mol).

The individual protomers of AdeJ adopt one of the three main conformational states that are referred to as access, binding, and extrusion (14–16). To date, three cryo-electron microscopy (EM) structures have been determined for AdeJ (14, 31), two of which are in ligand-bound states with fluorocycline TP-6076 (PDB ID 7RY3 [14], chain C) and the antibiotic ERV (PDB ID 7M4P [31], chain B) in the binding protomers and one apo structure (PDB ID 7M4Q [31]). We used all three structures for computational docking and removed the ligands from the two holo structures. We docked eight candidate inhibitors (**2**, **12**, **17**, **22**, **24**, **25**, **29**, and **30**) and substrates (EtBr, ERV, and TP-6076) to the Access, Binding, and Extrusion protomers at the entrance, PBP, and DBP sites (Fig. S4). In addition to WT AdeJ, we docked each compound to models of the E675A, R701A, and F178C mutants, which exhibit altered MPC_4_ values relative to WT for some of the eight compounds (Table 4; Fig. 6).

**TABLE 4:** Most favorable docking scores for candidate inhibitors and substrates docked to WT and selected mutants of AdeJ at the entrance, proximal (PBP), and distal (DBP) sites

Compound	Entrance		PBP		DBP	
	R701 (WT)	A701 (mutant)	E675 (WT)	A675 (mutant)	F178 (WT)	C178 (mutant)
2	−9.1	−8.7	−11.8	−12.2	−13.2	−12.9
12	−8.4	−7.8	−10.5	−10.2	−11.5	−10.6
17	−7.6	−7.6	−9.1	−8.7	−10.4	−9.5
22	−7.9	−7.9	−9.6	−9.5	−10.8	−10.3
24	−8.4	−8.2	−10.6	−10.8	−12.1	−10.3
25	−7.9	−7.7	−9.6	−9.9	−11.1	−10.3
29	−8.4	−7.8	−9.3	−9.6	−11.1	−10.3
30	−7.8	−7.6	−10.0	−9.9	−10.9	−10.5
EtBr	−7.7	−6.8	−10.7	−10.6	−10.3	−10.7
ERV	−7.2	−7.3	−10.1	−9.8	−9.3	−9.4
TP-6076	−7.4	−6.6	−9.0	−8.7	−9.7	−9.0

The overall most favorable docking scores correspond to compounds bound to the DBP (Table 4; Fig. S5A). The docking scores are more favorable for sites 675 (F-loop in the PBP) and 178 (DBP) than site 701 (entrance site). The binding protomer shows the most favorable scores among the protomers, with the extrusion and access protomers being less favorable (Fig. S5B). The general trend in docking scores at the three sites (i.e., weakest binding at the entrance and strongest at the distal site) suggests directionality in the ligand pathway from the exterior of the pump to the DBP where extrusion occurs, consistent with findings for AdeB in previous studies (32, 34).

Compound **2** consistently provides docking scores among the most favorable at the proximal and distal sites in any of the three protomers. Overall, the most favorable site and protomer for compound **2** are the DBP and binding, respectively, with a docking score of −13.2 kcal/mol for the WT transporter. The corresponding score for the F178C mutant is similar (−12.9 kcal/mol). Compound **24** also showed favorable docking scores at the proximal and distal sites in the access and binding protomers (e.g., −12.1 kcal/mol at the distal site in the binding protomer). The docking scores for these compounds were also somewhat more favorable than those of the substrates TP-6076, ERV, and EtBr (Fig. S5). The more favorable scores for the candidate inhibitors can be attributed to additional interactions made with DBP residues compared to the substrates. For example, additional stacking interactions are predicted between the naphthyl moieties of compound **2** and F616/F629. In addition, the phenolic ring interacts with Y327, whereas only stacking interactions between the *p*-fluorobenzaldehyde ring and the side chain of F616 are present in the ERV-bound cryo-EM structure (Fig. S7). In contrast, the docking scores for compounds **17** and **22** tend toward somewhat less favorable values (Table 4; Fig. S5).

We observed no major differences in docking scores and poses between the WT and corresponding mutants for any of the three mutations considered (Table 4; Fig. S8). Therefore, either the docking calculations are not sufficiently discriminatory or factors other than binding affinity are responsible for the observed changes in MPC_4_ values in the mutants compared to the WT. Other factors may include changes in the conformational dynamics of the pump, possibly involving the flexibility of the loops between the DBP and PBP. Such behavior has been noted previously for AcrAB-TolC, in which structural changes were found to be a major factor leading to antibiotic resistance in addition to ligand binding to the DBP (35, 36).

## DISCUSSION

EPIs are promising alternative therapeutics that have the potential to restore activities of antibiotics that are negatively affected by efflux in clinical antibiotic-resistant pathogens. In this study, we analyzed interactions of EPIs with the ligand translocation path of AdeJ by introducing site-specific substitutions and by analyzing the properties of the constructed AdeJ variants using a combination of antibacterial and kinetic assays. We found that substitutions in AdeJ strongly affect the antibiotic potentiating and efflux inhibitory activities of the analyzed EPIs, either enhancing their activities or leading to resistance against EPIs. Our results also validate AdeJ as a target of DAQ activity and suggest a possible mechanism of their action.

Among amino acid residues targeted for mutagenesis, four (F618, F178, V139, and F136) have been reported to form direct stacking and hydrophobic interactions with ERV and fluorocycline ligands in the DBP of AdeJ (14, 31). Surprisingly, the F618, V139, and F136 substitutions in these sites did not notably reduce the MICs of ERV and its analog TET (Table 1), as could be expected for the interactions required for the recognition and transport of these antibiotics. Furthermore, removal of aromatic rings in sites 136 and 618 even increased the MICs of ERV, TET, CHL, and norfloxacin, pointing to the increased efflux of these ligands and non-essentiality of these aromatic side chains in substrate binding and efflux by AdeJ.

In contrast, the substitution of F178 to cysteine reduced MICs of several antibiotics, compromised AdeJ-dependent efflux of fluorescent probes, and increased AdeJ sensitivity, specifically to naphthyl- and certain biphenyl-substituted EPIs. This result is strikingly different from the effect of analogous F178C substitution in the AdeB efflux pump, where such substitution enhanced efflux of select substrates (32). In this light, the finding that the analyzed EPIs inhibit the AdeJ F178C variant further implies that, unlike previously reported EPIs such as MBX3132 and ABI-PP that form stacking contacts with F178 in *E. coli* AcrB (23) and *P. aeruginosa* MexB (22), respectively, DAQs do not need F178 of AdeJ for their efflux inhibitory activity. These results suggest that, contrary to structural data, F178 and possibly other aromatic residues in the hydrophobic trap of RND efflux pumps are not engaged in ligand recognition and play a context-dependent role in conformational transitions during transport. In agreement with this concept, molecular docking calculations showed that F178 does not interact with DAQs (Fig. 6), and its substitution does not affect the docking scores of these compounds (Table 4). Since the observed changes in antibacterial activities are specific only to select substrates, such as azithromycin and zeocin in the case of the AdeB F178C variant or EtBr and NOV and certain DAQs with the AdeJ F178C variant, ligands bound somewhere else likely participate in these conformational transitions and can either increase or decrease the transporter’s turnover rates and therefore efficiency of EPIs.

To reach the DBP, ligands are expected to be attracted into the transporter’s entrance and then move into the PBP. Our results show that substitutions in amino acid residues located in the entrance cleft (R701) and the F-loop (E675) lead to resistance against DAQs. The molecular mechanism underlying this resistance phenotype is not immediately clear. The entrance of the AdeJ periplasmic cleft is surrounded by residues M666, L668, R701, R718, and T831, and the affinity to these residues is likely to play a role in substrate specificity and selectivity. The positively charged character of the entrance suggests that negatively charged and zwitterionic molecules could be attracted to it and that the R701A mutant might thus affect recognition of substrates and EPIs, although in the docking poses, this residue makes no contacts with DAQ ligands (Fig. 6) nor in the ligand-bound structures of the homologous pumps (13). This suggests possible kinetic control of binding of these ligands. We also found that the R701A substitution has no effect on MICs of NOV (pKa 4.3), which is one of the best substrates of AdeJ (Table 1) (9). This substitution also has no effect on the DAQ-mediated potentiation of the antibacterial activity of this antibiotic. In contrast, R701A increases MICs of ERY, TET, and EtBr and reduces the inhibitory effect of EPIs in combination with these positively charged compounds and only for analogs **29** and **30** with the electron-accepting/donating methoxy substitution. These results can be reconciled by the structural data that showed a salt bridge between R701 and E723 in the WT AdeJ. This salt bridge would be broken in the R701A mutant, perhaps opening the negative charge of E723 to interactions with substrates and DAQs.

A conserved F-loop (residues 671–680) connects the cleft entrance to the proximal drug-binding site. This F-loop also forms the bottom section of the proximal site. Unlike R701A, the substitution of E675 with alanine does not affect MICs of NOV, ERY, TET, and EtBr but provides resistance against all four biphenyl DAQs in combination with these four substrates. The E675A variant, however, protects better against zeocin, the largest antibiotic in the tested set, and is less efficient against the detergent SDS (Table 1). Previous studies (13, 31) and our docking results (Fig. 6B) show that ligands move through the interface between PBP and DBP guided by F- and G-loops. In the WT AdeJ structure, E675 forms hydrogen bonds with the backbone of I38 and A39, which likely restrict the mobility of the F-loop and impose more selectivity during the transition of ligands from the entrance into the PBP. The substitution of E675 with alanine would break these stabilizing interactions, leading possibly to less restrictive translocation of ligands along the substrate path for efflux. By this logic, biphenyl DAQs have a kinetic advantage over antibiotics when captured by the AdeJ E675A variant, and their more efficient expulsion could explain the higher concentrations of EPIs needed to potentiate antibiotics, as seen in the higher MPC_4_ values in combination with antibiotics.

## MATERIALS AND METHODS

### Construction of mutants

The bacterial strains and plasmids used in this study are shown in Table S6. All single mutations in the *adeJ* gene were constructed by the Q5 Site-Directed Mutagenesis kit (NEB) using pIL131 (pTJ1-*adeIJK*) as the template. The corresponding mutations and the lack of undesired mutations in genes or plasmid were verified by plasmid sequencing (Plasmidsaurus). These plasmids were inserted into AbΔ3 and AbΔ3-pore strains, as described previously (9).

### Microbiological assays

All bacterial cultures were grown in Luria-Bertani (LB) broth complemented with 100 μg/mL trimethoprim, if needed, at 37°C. The MICs of different antibiotic classes for AdeJ mutant strains were determined in 96-well microtiter plates at 37°C in LB medium supplemented with 1% L-arabinose. MICs were analyzed using a twofold broth dilution method (37). The lowest antimicrobial concentration that inhibits visible growth was defined as its MIC.

MPC_4_ of the AdeJ efflux inhibitors was calculated, as described previously (38). MPC_4_ was done in AdeJ variants for previously published EPIs (Fig. 3) (30). Interactions between the substrates and the inhibitors were assessed by a checkerboard titration assay as described before (30). Briefly, the checkerboard 96-well plates comprised efflux inhibitor test compound dilutions (200.0–1.56 μM) made in each row and antibiotic dilutions (NOV, TET, or ERY) made in each column. Plates were incubated at 37°C for 20 h. The absorbance at 600 nm was measured on a Tecan Spark 10M multimode microplate reader, and the inhibitory concentrations were used for the calculation of fractional inhibitory concentration index values.

### Protein expression analyses

SDS-polyacrylamide gels and immunoblotting analyses were used to analyze the expression level of mutated AdeJ proteins in the AbΔ3 and AbΔ3-pore cells. One milliliter of cells with OD_600_ = 1 was collected after 3 h of induction with 1% arabinose and lysed. The lysate was analyzed by 10% SDS-PAGE, transferred to polyvinylidene difluoride membranes for immunoblotting with primary polyclonal anti-AdeJ antibodies (Thermo Fisher Scientific) and secondary alkaline phosphatase-conjugated antirabbit immunoglobulin antibody (Sigma). The polyclonal anti-AdeJ antibody production was done as described before (32). The 5-bromo-4-chloro-3-indolylphosphate and nitroblue tetrazolium substrates were used to visualize the bands.

### Efflux assays

EtBr and NPN uptake assays were performed in a temperature-controlled microplate reader (Tecan Spark 10M multimode microplate reader) equipped with a sample injector in fluorescence mode, as described previously (9, 30). Additionally, the intracellular accumulation of EtBr (5 µM final concentration) in the presence (50.0–3.125 μM) of selected efflux inhibitors was measured. For all experiments, overnight cells were subcultured (1:50), induced with 1% arabinose in 1 h, and grown for an additional 3 h at 37°C. Then, cells were collected by centrifugation, washed in HMG buffer (50 mM HEPES-KOH, pH 7.0, 1 mM MgSO_4_, and 0.4% glucose), and adjusted to an OD_600_ of 1.0. Fluorescence of EtBr was monitored at *λ*_ex_ of 480 nm and *λ*_em_ of 610 nm, and NPN at *λ*_ex_ of 350 nm and *λ*_em_ of 405 nm at a gain of 75 for 10 min. Fluorescence intensities were plotted against time in Microsoft Excel and normalized to the emission before cells were added. These data were imported into MATLAB (MathWorks) to be fitted to a simple exponential equation in the form of *F* = *A*1 + *A*2[1-exp(−kt)] (33). All measurements were done at least two times in duplicate.

### Molecular docking

SMILES strings were converted to SDF format with RDKit. The program xTB was then used to optimize the geometry of each molecule at the GFN2-xTB level of theory (39). The analytical linearized Poisson-Boltzmann (ALPB) implicit solvation model (40) was used during geometry optimization. Meeko was used to generate PDBQT files for each molecule.

Models of AdeJ with selected residue mutations (i.e., F178C, E675A, and R701A) were generated with the *fixbb* application in Rosetta version 3.9 (41). For the WT and each mutant, a docking box with dimensions of 25 × 25 × 25 Å was centered at the Cα of the selected residue (Fig. S4). All docking calculations were performed with AutoDock Vina version 1.2.5 (42, 43) with the default exhaustiveness setting of 8. To validate our protocol, we first redocked the substrates TP-6076 and ERA to their corresponding cryo-EM structures (PDB ID: 7RY3 and 7M4P, respectively). We obtained favorable docking scores and low root mean square deviation values relative to the corresponding cryo-EM poses (TP-6076/7RY3: −10.3 kcal/mol, 0.8 Å, and ERA/7M4P: −9.1 kcal/mol, 1.4 Å) (Fig. S6). Candidate inhibitors **2**, **12**, **17**, **22**, **24**, **25**, **29**, and **30** and efflux substrates EtBr, ERV, and TP-6076 were then docked to the mutant and WT AdeJ structures.

## SUPPLEMENTAL MATERIAL

Supplemental material aac.00090-25-s0001.pdf Tables S1 to S6; Fig. S1 to S8.

ASM does not own the copyrights to Supplemental Material that may be linked to, or accessed through, an article. The authors have granted ASM a non-exclusive, world-wide license to publish the Supplemental Material files. Please contact the corresponding author directly for reuse.
