# Supplementary material for: Mutations in the proximal binding site and F-loop of AdeJ confer resistance to efflux pump inhibitors
Source: Antimicrob Agents Chemother. 2025 Jul 8;69(8):e00090-25. doi: 10.1128/aac.00090-25 (PMC12326959; doi:10.1128/aac.00090-25)
Supplement: Supplemental material — Tables S1 to S6; Fig. S1 to S8. [file aac.00090-25-s0001.pdf]

Supplemental Information for

Mutations in proximal binding site and F-loop of AdeJ confer resistance to efflux pump inhibitors

Aysegul Saral Sariyer[1,2], Inga V. Leus[1], Rushikesh Tambat[1], Mithila Farjana[1], Marcela Olvera[1], Shalini J. Rukmani[3], Emrah Sariyer[1,2,4], Jeremy C. Smith[3], Jerry M. Parks[3], John K. Walker[5,6] and Helen I. Zgurskaya[1,*]

[1]University of Oklahoma, Department of Chemistry and Biochemistry, Norman, OK, USA

[2] Artvin Coruh University, Department of Nutrition and Dietetics, Faculty of Health Sciences, 08000 Artvin, Turkey

[3] Bioscience Division, Oak Ridge National Laboratory, 1 Bethel Valley Road, Oak Ridge, TN, 37831, USA

[4]Artvin Coruh University, Vocational School of Health Services, Medical Laboratory Techniques, 08000, Artvin, Turkey

[5] Saint Louis University School of Medicine, St. Louis, MO, USA

[6]Saint Louis University, Department of Chemistry, St. Louis, MO, USA

[*]Corresponding authors: elenaz@ou.edu

Table S1. Quantitation of relative intensities in immunoblotting analysis of AdeJ expression from efflux-deficient AbΔ3 cells carrying an empty vector and the indicated plasmid-borne AdeJ variants and corresponding hyperporinated derivatives.

**Strains**	**% Intensity**	
	**Pore (-)**	**Pore (+)**
ATCC17978	23.46	31.5
Vector	-	-
WT	100	100
G721I	150.28	154.85
R701A	126.85	212.92
E675A	160.19	134.28
F618A	96.36	207.26
F178C	83.09	171.56
V139C	103.14	191.42
F136A	88.37	210.86
A134I	109.55	181.08
N81A	64.73	206.69

Note: The image acquisition and quantitation were done using Quantity one® 1-D analysis software.

Table S2. Minimal inhibitory concentrations (MICs, μg/mL) of substrates in the hyperporinated wild type, efflux-deficient *A. baumannii* strains and the efflux-deficient strain producing the indicated AdeJ variants.

Strains	Location	MICs (μg/mL)									
		**ERY**	**NOV**	**ZEO**	**CIP**	**NOR**	**ERV**	**TET**	**CHL**	**SDS**	**EtBr**
Vector	-	0.625	<0.02	0.25	0.008	0.125	0.016	0.016	32	16	1
WT	-	10	8	8	0.625	4	0.25	0.5	128	>1024	32
E675A	F-loop	5	8	4	0.125	4	0.25	0.25	64	512	16
N81A	PBP	10	4	8	0.125	8	0.25	0.5	128	>1024	32
R701A		10	4	2	0.25	8	0.25	0.25	128	>1024	32
G721I		10	4	2	0.125	4	0.25	0.125	64	512	32
F618A	G-loop	10	4	4	0.125	8	0.25	0.5	256	>1024	32
A134I	DBP	10	2	2	0.125	4	0.125	0.25	128	>1024	32
F136A		20	2	2	0.25	8	0.5	0.5	256	>1024	64
V139C		5	4	4	0.125	4	0.25	0.25	128	512	16
F178C		2.5	1	0.5	0.016	1	0.25	0.25	32	256	4

Abbreviations in this and other tables: SDS, sodium dodecyl sulfate; ERY, erythromycin; EtBr, ethidium bromide; NOV, novobiocin; ZEO, zeocin; CIP, ciprofloxacin; NOR, norfloxacin; ERV, eravacycline; TET, tetracycline; CHL, chloramphenicol; PBP, proximal binding pocket; DBP, distal binding pocket.

Table S3. MPC_4 of benzoyl-substituted DAQs in combinations with indicated antibiotics in AbΔ3-pore strain producing the indicated AdeJ variants.

**Strains**	**MPC_4 (µM)**											
	**NOV**			**ERY**			**EtBr**			**TET**		
	**12**	**17**	**22**	**12**	**17**	**22**	**12**	**17**	**22**	**12**	**17**	**22**
WT	12.5	6.25	6.25	6.25	25	25	12.5	25	12.5	25	50	50
E675A	12.5	12.5	6.25	12.5	25	25	12.5	50	12.5	50	50	25
N81A	25	12.5	12.5	6.25	12.5	12.5	6.25	25	12.5	25	50	50
R701A	6.25	12.5	6.25	6.25	25	25	12.5	12.5	12.5	25	50	50
G721I	6.25	12.5	6.25	6.25	50	25	25	50	12.5	25	50	50
F618A	6.25	12.5	12.5	3.125	25	25	12.5	25	25	25	50	50
A134I	6.25	12.5	12.5	12.5	50	50	25	25	12.5	25	50	50
F136A	25	12.5	12.5	6.25	12.5	25	12.5	25	12.5	12.5	25	25
V139C	3.125	12.5	6.25	6.25	25	25	12.5	25	12.5	25	50	50
F178C	6.25	6.25	3.125	3.125	25	25	6.25	25	12.5	25	50	25

Table S4. MICs and MPC_4 of EPIs in combinations with ZEO and EtBr in AbΔ3 and AbΔ3-pore strain producing the indicated AdeJ variants.

Strains	Pore	MICs (µM)				ZEO MPC_4 (µM)			EtBr MPC_4 (µM)			
		**2**	**24**	**29**	**30**	**2**	**29**	**30**	**2**	**24**	**29**	**30**
WT	+	>200	>200	>200	>200	25	12.5	25	25	50	50	25
	-	>200	ND	>200	>200	25	12.5	25	ND	ND	ND	ND
F178C	+	>200	>200	>200	100	**6.25**	**3.125**	**6.25**	**6.25**	50	**12.5**	**6.25**
	-	>200	ND	>200	100	**12.5**	**6.25**	**12.5**	ND	ND	ND	ND
E675A	+	>200	>200	>200	>200	25	**50**	**100**	**25**	**200**	**200**	**100**
	-	>200	ND	>200	>200	25	**50**	**100**	ND	ND	ND	ND
R701A	+	>200	50	>200	>200	25	**50**	**100**	**25**	50	**200**	**200**
	-	>200	ND	>200	>200	25	**50**	**100**	ND	ND	ND	ND

Table S5. MICs of indicated antibiotics in the presence and absence of EPIs for AbΔ3-pore strain producing the indicated AdeJ variants.

Conc. of EPIs (μM)	MICs (μg/mL) of antibiotics in the presence of EPIs							
	NOV				ERY		TET	
	Compound 24		Compound 2		Compound 29		Compound 30	
	AdeIJK	E675A	AdeIJK	F178C	AdeIJK	R701A	AdeIJK	E675A
200	<0.0625	0.25	<0.0625	<0.0625	<0.0625	0.25	0.0078	0.0625
100	0.125	0.5	<0.0625	<0.0625	0.125	1	0.0312	0.125
50	0.25	1	0.125	<0.0625	0.25	2	0.0625	0.125
25	0.5	2	0.25	<0.0625	0.5	4	0.125	0.25
12.5	0.5	4	1	0.125	2	4	0.125	0.25
6.25	1	4	4	0.25	2	4	0.25	0.25
3.125	2	8	4	0.5	4	8	0.25	0.25
1.56	4	8	8	2	8	8	0.25	0.25
0	8	8	8	2	8	8	0.25	0.25

Table S6. Strains and plasmids used in this study.

Strain	Relevant genotype
JWW30 (Ab WT)	ATCC 17978, spontaneous variant resistant to 100 µg/mL streptomycin
IL125 (Ab WT-pore)	*A.baumannii* JWW30 *att*Tn*7*::mini-Tn*7*T-Kan^r-*araC*-P$_{BAD}$-FhuA
IL119 (AbΔ3)	*A. baumannii* JWW30 *ΔadeAB ΔadeFGH ΔadeIJK*
IL139 (AbΔ3-pore)	*A.baumannii* IL119 *att*Tn*7*::mini-Tn*7*T-Kan^r-*araC*-P$_{BAD}$-FhuA
IL122 (AbΔ3(Vector))	*A.baumannii* IL119 *att*Tn*7*::miniTn7T-Tp^r-*araC*-P$_{BAD}$-MCS carrying pTJ1
IL161 (AbΔ3(Vector)-pore)	*A.baumannii* IL139 *att*Tn*7*::miniTn7T- Kan^r -*araC*-P$_{BAD}$-FhuA carrying pTJ1
IL142 (AbΔ3(AdeIJK))	*A. baumannii* IL119 attTn7::mini-Tn7T-Tp^r-*araC*-P$_{BAD}$-*adeIJK* carrying pTJ1-*adeIJK*
IL148 (AbΔ3(AdeIJK)-pore)	*A.baumannii* IL139 *att*Tn*7*::mini-Tn*7*T-Kan^r-*araC*-P$_{BAD}$-FhuA carrying pTJ1-*adeIJK*
**Plasmids**	
pIL131 (pTJ1-*adeIJK*)	pUC18T-mini-Tn*7*T-Tp-*araC*-P$_{BAD}$-*adeIJK*, Amp^r, Tp^r
pET-21 a (+)	T7*lac,* T7-Tag (N), His-Tag (C), Amp^r
pIL152 (pET21- AdeJ)	AdeJ inserted between NdeI and XhoI; Amp^r

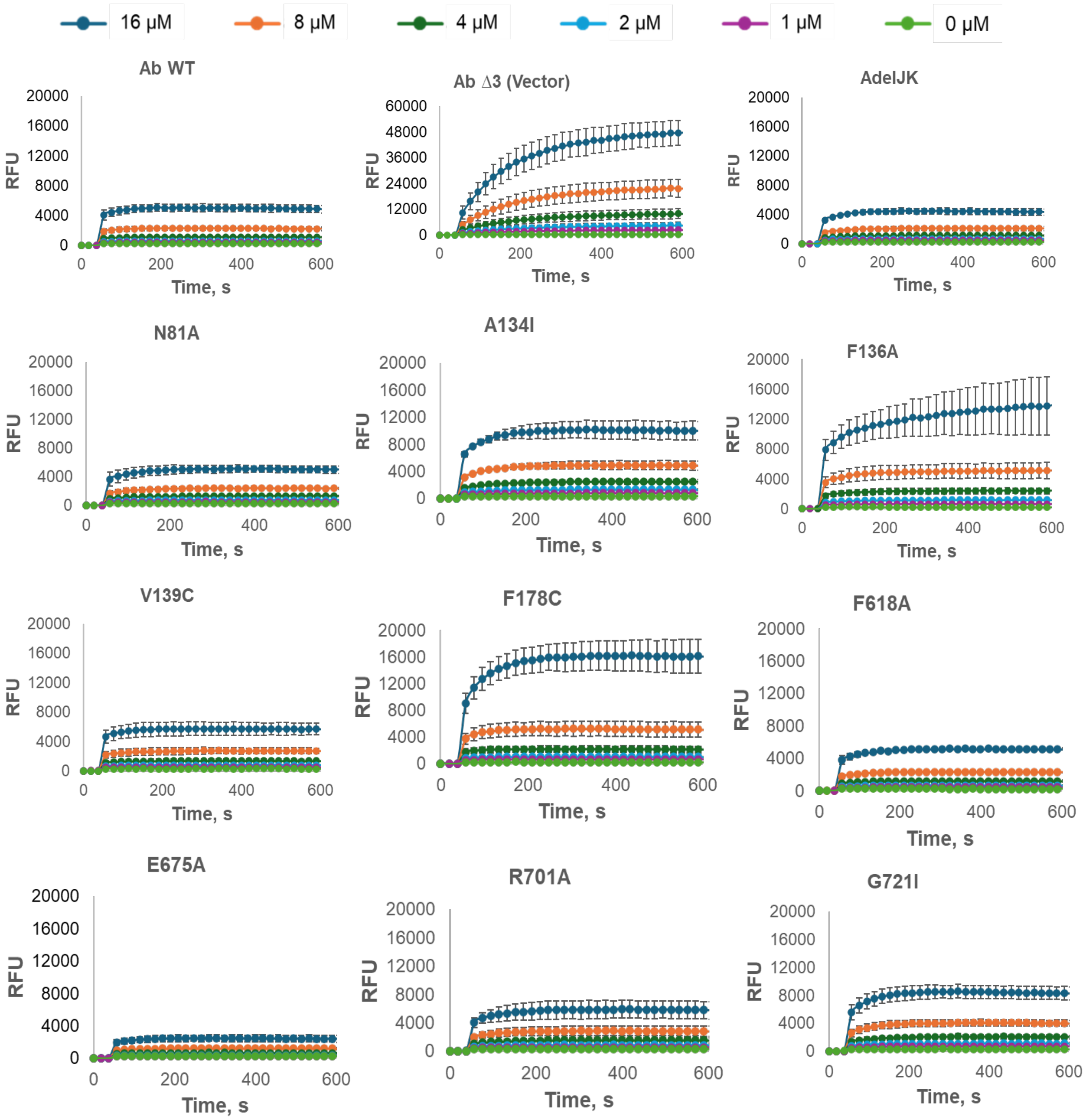

Figure S1. Intracellular uptake of the *N*-phenyl-naphthylamine (NPN) in *A. baumannii* wild-type and Δ3 cells with its variants. Data represent real-time kinetics of changes in NPN fluorescence (0 µM-16 µM final external concentration). Each time point represents the average of at least two biological replicates with two technical repeats ± standard deviation (SD).

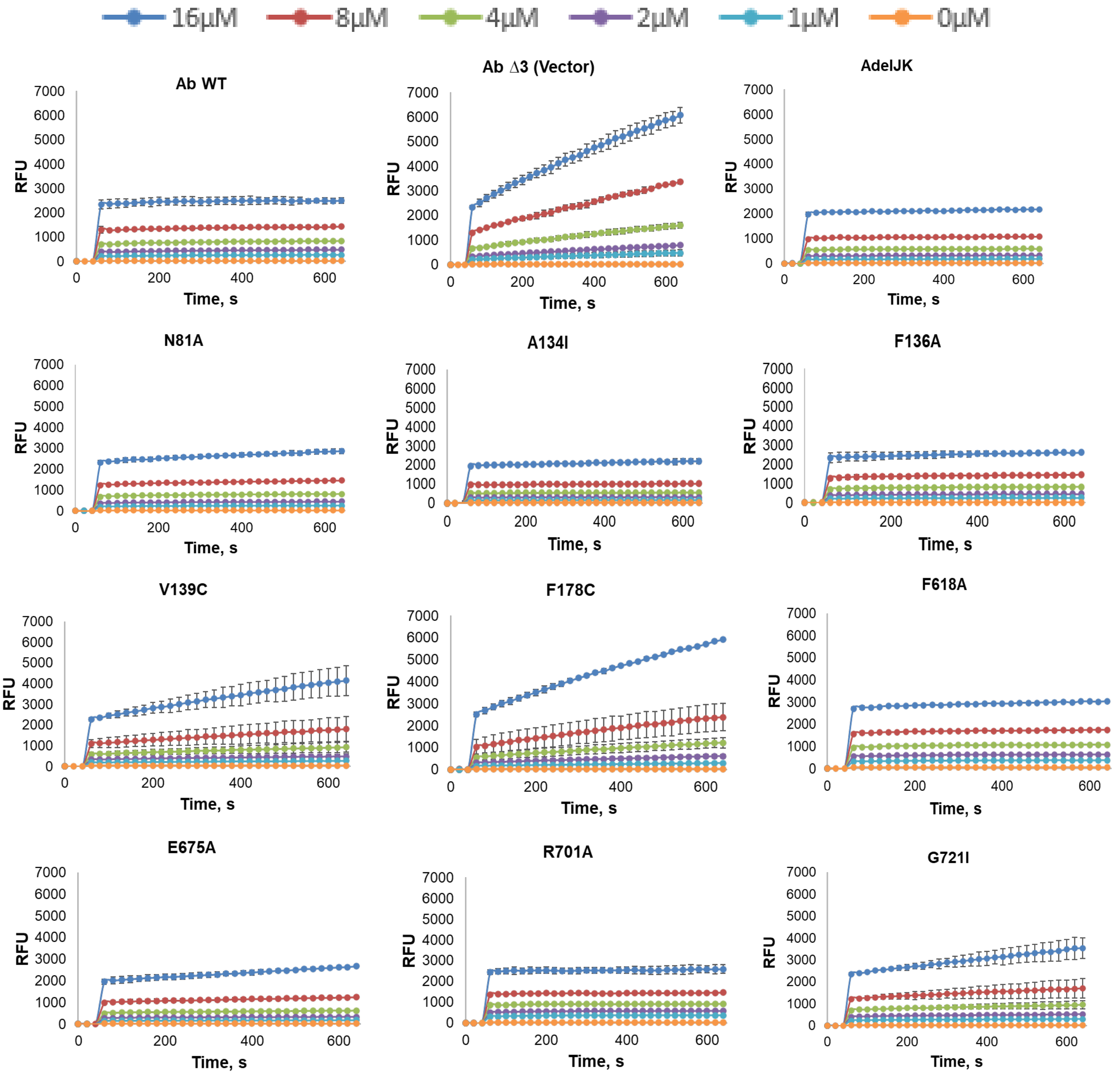

Figure S2. Intracellular uptake of the ethidium bromide (EtBr) in *A. baumannii* wild-type and Δ3-pore cells with its variants. Data represents real-time kinetics of changes in EtBr fluorescence (0 µM-16 µM final external concentration). Each time point represents the average of two biological replicates with two technical repeats ± SD.

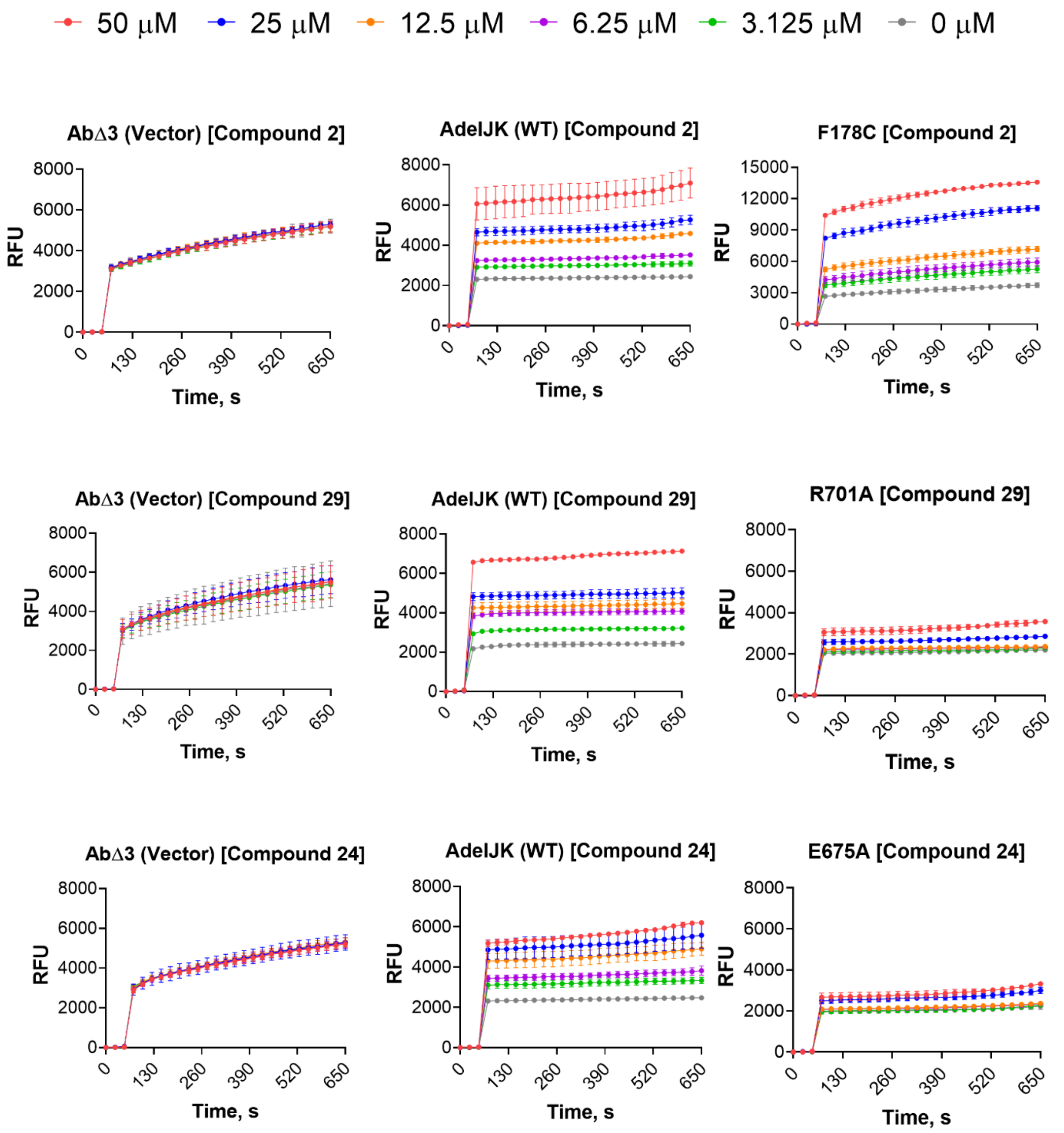

Figure S3. Intracellular uptake of the fluorescent probe EtBr in the presence and absence of EPIs. Data represents real-time kinetics of changes in EtBr fluorescence (4 µM final external concentration) in the presence of compound 2, 29, and 25 (0 µM-50 µM final external concentration). Each time point represents the average of two biological replicates with two technical repeats ± SD.

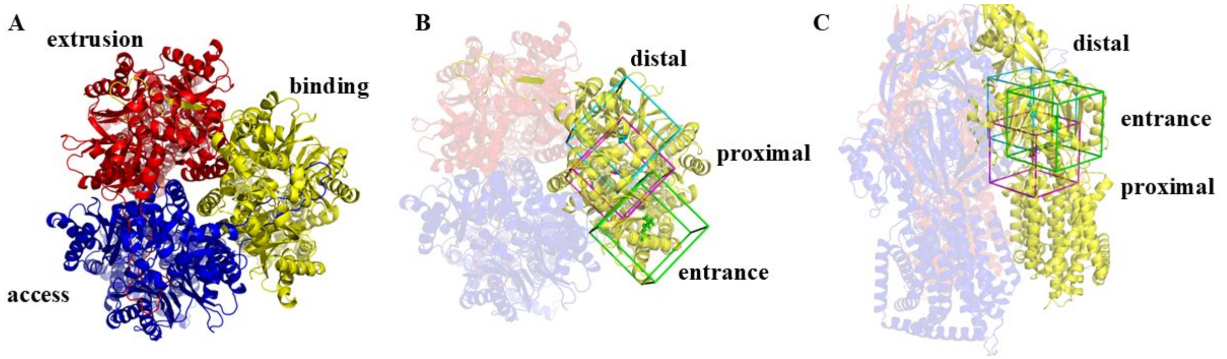

Figure S4. Structure of AdeJ (PDB ID: 7RY3) from[1]. (**A**) Top view of the three pump conformations of AdeJ, (**B**) Docking boxes for three main ligand binding sites (top view), (**C**) Alternate, lateral view of docking boxes in B.

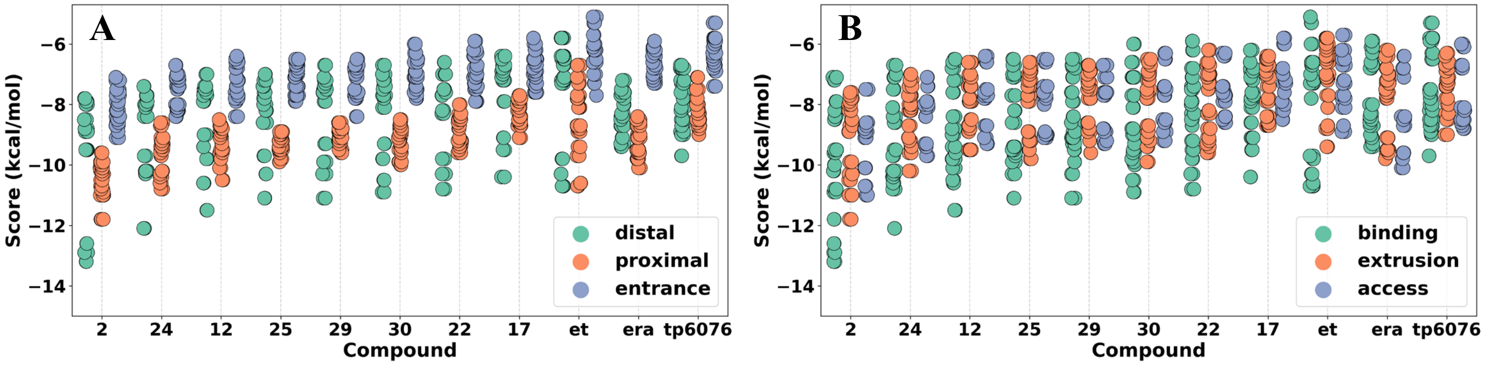

Figure S5. Docking scores for candidate inhibitors and substrates (EtBr, ERV, and TET analogue TP-6076[1]) to the wild type and mutants in AdeJ structures (PDB ID 7RY3[1], 7M4P, 7M4Q[2]) showing distribution of scores by (**A**) sites (distal: F178/C178, proximal: E675/A675, and entrance: R701/A701), and (**B**) protomer states (binding, extrusion, and access).

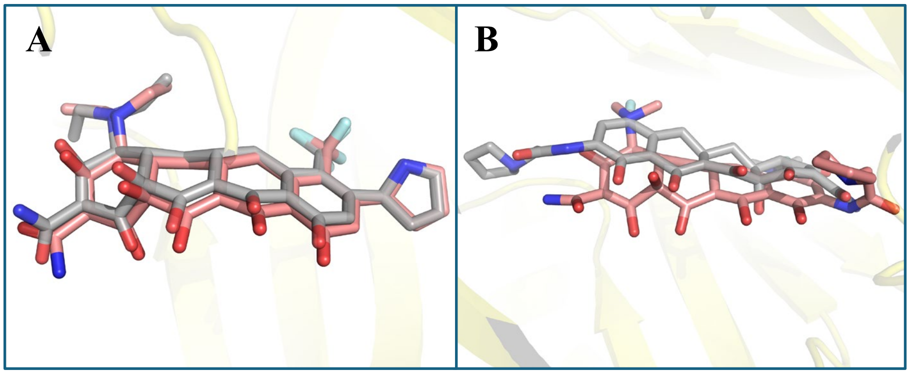

Figure S6. Superposition of re-docked substrates onto the corresponding cryo-EM poses of AdeJ. (**A**) TP-6076 (PDB ID: 7RY3) with a docking score of -10.3 kcal/mol and RMSD of 0.8 Å. (**B**) ERV (PDB ID: 7M4P) with a docking score of -9.1 kcal/mol and RMSD of 1.4 Å. Color scheme: cryo-EM pose (grey) and re-docked pose (pink).

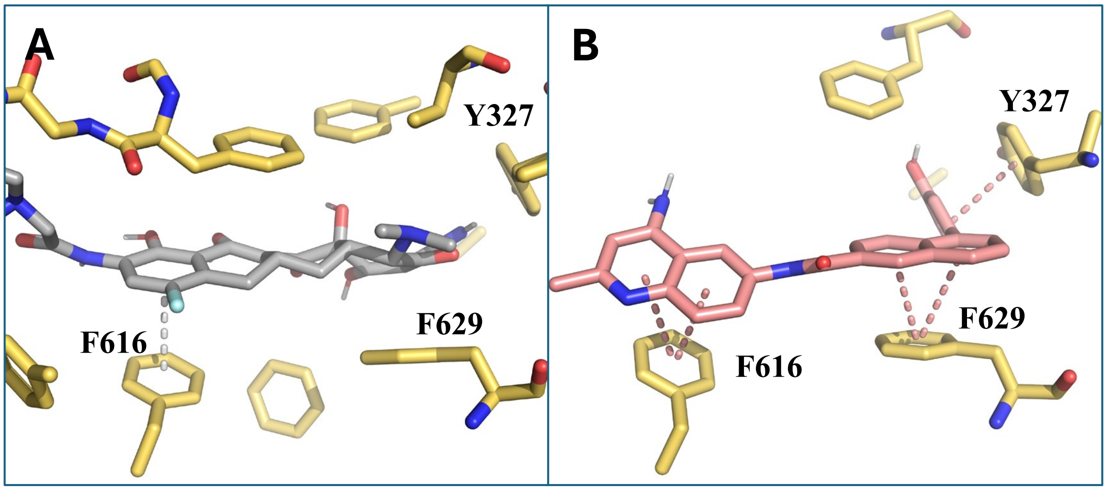

Figure S7. Interactions between substrate/candidate inhibitors with the AdeJ residues (PDB ID: 7M4P) (**A**) stacking between the p-fluorobenzaldehyde ring of ERA (gray) and F616 (**B**) stacking between the napthyl rings in compound 2 (pink) with F616 and F629 residues and the phenolic ring with Y327.

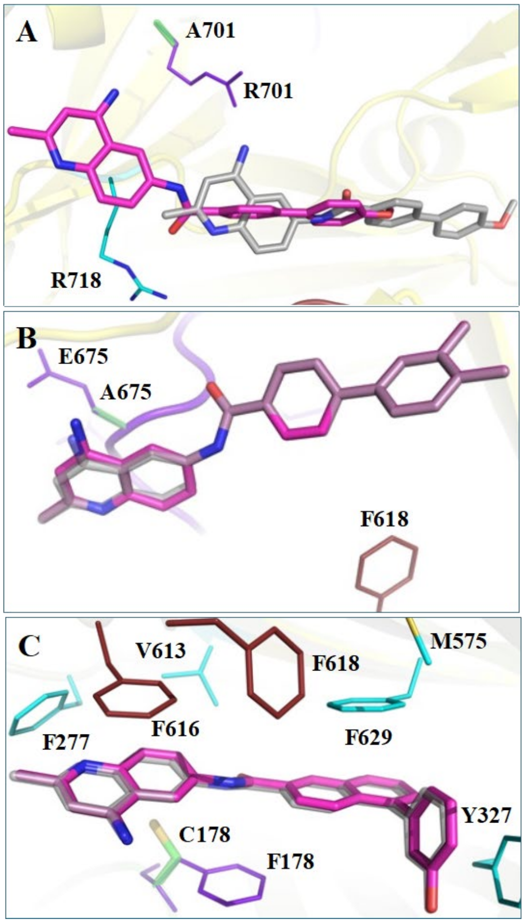

Figure S8. Superposition of docked poses of selected ligands to the wild type and mutants in the binding protomer of AdeJ (PDB ID 7RY3, chain C). (**A**) Compound **29** bound at the entrance site of AdeJ (R701/A701) (**B**) Compound **24** bound at the proximal site (E675/A675) (**C**) Compound **2** bound at the distal site (F178/C178). Ligands docked to the wild type are shown in grey. Ligands docked to the mutant are shown in magenta. Wild type and mutant side chains at positions 701, 675, and 178 are shown in purple and green sticks, respectively. Side chains of residues in the F-loop and neighboring residues are shown in brown and cyan, respectively.

Supplemental References

1. Morgan, C. E.; Zhang, Z.; Bonomo, R. A.; Yu, E. W., An analysis of the novel fluorocycline TP-6076 bound to both the ribosome and multidrug efflux pump AdeJ from Acinetobacter baumannii. *MBio* **2022,** *13* (1), e03732-21.

2. Zhang, Z.; Morgan, C. E.; Bonomo, R. A.; Yu, E. W., Cryo-EM Determination of Eravacycline-Bound Structures of the Ribosome and the Multidrug Efflux Pump AdeJ of Acinetobacter baumannii. *mBio* **2021,** *12* (3), e0103121.
